# Conversational Agents for Asthma and Chronic Obstructive Pulmonary Disease Management: Scoping Review

**DOI:** 10.2196/98338

**Published:** 2026-09-29

**Authors:** Nurfaizah Binte Hassan, Tiang Peng Teo, Mei Fong Liew, Wee Hian Tan, Wenjie Wu, Timotius Marvin Tan, Siti Aisah Binte Mustafah, Deborah Wan Qin Poh, Hong-Gu He, Joanne Huiyi Khor, Genevieve Mei Yun Tan, Si Qi Yoong, Ying Jiang

**Affiliations:** 1 Department of Nursing, National University Hospital, National University Health System, Singapore, Singapore; 2 Alice Lee Centre for Nursing Studies, Yong Loo Lin School of Medicine, National University of Singapore, Block MD6, Level 5, 14 Medical Drive, Singapore, 117599, Singapore, +65 6516 7791; 3 Division of Respiratory and Critical Care Medicine, Alexandra Hospital, National University Health System, Singapore, Singapore; 4 Division of Respiratory and Critical Care Medicine, Department of Medicine, National University Hospital, National University Health System, Singapore, Singapore; 5 National University Polyclinics, National University Health System, Singapore, Singapore; 6 Group Chairman Medical Board Office, National University Health System, Singapore, Singapore; 7 National University Health System, Singapore, Singapore; 8 Duke-NUS Medical School, Singapore, Singapore

**Keywords:** conversational agents, chatbot, AI, obstructive lung diseases, asthma, COPD, chronic obstructive pulmonary disease

## Abstract

**Background:**

Asthma and chronic obstructive pulmonary disease (COPD) affect more than 650 million people worldwide and remain leading causes of disability, with a rising burden as populations age. Conversational agents (CAs) may offer a more interactive alternative. However, the evidence in obstructive lung disease has not been mapped.

**Objective:**

The aim of this study is to map the literature on CA use in asthma and COPD, to describe the roles they have been designed to perform, the outcomes that have been measured, and to map the study designs and methods characterizing the current evidence.

**Methods:**

A scoping review was conducted following the Arksey and O’Malley framework. A total of 9 databases (CINAHL, CENTRAL, Embase, IEEE Xplore, PubMed, ProQuest, Scopus, Web of Science, and Google Scholar) were searched from January 1, 2014, to February 22, 2025. Data were synthesized using inductive content analysis and organized via the Patterns, Advances, Gaps, Evidence for Practice, and Research Recommendations (PAGER) framework. Reporting followed PRISMA-ScR (Preferred Reporting Items for Systematic Reviews and Meta-Analyses extension for Scoping Reviews) guidelines.

**Results:**

A total of 6275 records were screened, and 16 reports from 15 studies were included. Studies reported CAs being used for information provision and patient education, health data collection, and emotional or motivational support. Only 4 studies measured direct clinical outcomes (eg, asthma control or medication adherence). Reported usability and satisfaction findings were mixed, with recurring concerns about conversational flow, responsiveness, trust, and personalization.

**Conclusions:**

The evidence base for CAs in asthma and COPD remains in early developmental stages. It consists mainly of small feasibility, developmental, and pilot studies, providing limited evidence on whether CAs improve clinical outcomes. Current evidence can mainly describe the roles CAs have been designed to perform and the user-experience factors that influence engagement. Adequately powered, longitudinal trials using clinically meaningful and standardized endpoints are required before effectiveness can be assessed. Future development should place patients, as the end users, at the center of co-design, with clinicians, including nurses, involved to ensure clinical relevance and safe integration into care.

## Introduction

Asthma and chronic obstructive pulmonary disease (COPD) are among the most prevalent chronic respiratory diseases globally [1], collectively affecting over 650 million individuals, with prevalence projected to increase substantially by 2050 [1]. The Global Burden of Disease Study 2021 [2] estimated that 213.4 million people worldwide were living with COPD. Smoking accounted for the largest share of COPD-related disability-adjusted life years (34.8%), followed by ambient particulate matter pollution (22.2%) and household air pollution from solid fuels (19.5%). Although age-standardized rates of prevalence, mortality, and disability-adjusted life years have declined since 1990, the absolute burden continues to rise because of population growth and aging [2]. Characterized by chronic airway inflammation and airflow limitation, both diseases contribute significantly to morbidity, mortality, and health care usage. Beyond physical symptoms, patients frequently experience anxiety and depression, which exacerbate symptom perception and disease severity, further diminishing quality of life [3]. This psychosocial burden, coupled with rising health care costs, underscores the need for holistic management and innovative strategies to enhance disease monitoring and patient engagement.

Mobile health (mHealth) has become increasingly prominent in chronic respiratory disease management by improving access to care and patient convenience [4]. mHealth tools, such as medication reminders, symptom trackers, and educational resources, support adherence, self-management, and clinical outcomes [5]. However, these benefits are modest. A systematic review and meta-analysis of 17 randomized trials of digital health interventions in COPD involving 2027 participants found improved quality of life and self-efficacy, but no significant effect on 6-minute walk distance, emergency department attendance, or hospital admission [6]. Sustained engagement remains a major challenge: pooled dropout across app-based chronic disease interventions is approximately 43% (95% CI 29%-57%), increasing to 49% (95% CI 27%-70%) in observational settings [7]. These interventions often lack interactive, human-like features that sustain motivation and emotional connection, potentially limiting long-term engagement [8]. While mHealth facilitates remote monitoring and education, it generally falls short of providing real-time decision support during acute exacerbations [9], as most systems operate reactively, issuing alerts only after problems arise [10]. These limitations underscore the need for more intelligent, responsive, and personalized digital solutions for managing obstructive lung disease.

Conversational agents (CAs), or chatbots, are dialog systems that interact with users via natural language (written or spoken) [11]. A seminal example is ELIZA (Weizenbaum), developed in 1966 to emulate a psychotherapist’s conversational style [12], which catalyzed decades of innovation in conversational technologies. By the mid-2010s, advancements in natural language processing (NLP) and AI ushered in a transformative era, often described as the fourth wave of chatbot evolution. This era saw the introduction of voice-based CAs such as Siri (Apple Inc.), Alexa (Amazon.com, Inc.), and Google Assistant (Google LLC), which accelerated the adoption of conversational technologies [13]. Today, a wide spectrum of CAs exists, tailored to diverse purposes across various sectors (Multimedia Appendix 1). In health care, CAs offer significant potential to overcome the limitations of existing mHealth interventions. By leveraging NLP to simulate human-like conversations, CAs provide automated, round-the-clock responses, thereby enhancing access to care and continuity of care [14].

More recently, large language models (LLMs) have become widely accessible, and patients may use them to ask questions about their health without guidance from a doctor or health care provider. Early evaluations in respiratory care suggest such responses are often accurate but variable in readability and reliability [15-17]. This unsupervised use makes it more important to understand what purpose-built CAs for asthma and COPD have been designed to do, how they have been evaluated, and what evidence supports their use. This review therefore focuses on agents developed, adapted, or deployed for respiratory care.

To the authors’ knowledge, this is the first scoping review specifically examining the use of CAs in the management of obstructive lung diseases, as previous reviews have covered a broad range of chronic conditions and are not condition-specific, and respiratory-specific findings are diluted [8,18,19]. Methodological constraints were common, including restricted database searches, lack of reference list screening, and exclusion of non–open-access studies [18]. A total of 2 reviews are now outdated [20,21], reducing their relevance in the rapidly evolving field of AI and digital health. This review fills gaps by consolidating evidence on asthma and COPD and exploring how CAs support respiratory disease management. A scoping review was chosen because the field is still emerging and highly heterogeneous. This review therefore aims to clarify the types of technologies currently described as CAs in asthma and COPD, identify which roles and outcomes have been or are less explored, and map the study designs and methods characterizing the current evidence.

## Methods

### Overview

The use of CAs in chronic respiratory diseases is still emerging, with few robust experimental studies published to date. Therefore, a scoping review was conducted to map current and emerging literature. The review followed the Arksey and O’Malley framework: identifying the research question; identifying and selecting relevant studies; charting the data; and collating, summarizing, and reporting the results [22]. Additionally, the review adheres to the PRISMA-ScR (Preferred Reporting Items for Systematic Reviews and Meta-Analyses extension for Scoping Reviews) guidelines [23] (Checklist 1) and was prospectively registered on the Open Science Framework [24].

### Stage 1: Identifying the Research Question

This review aims to answer the following questions:

1. How are CAs (concept) currently used to support individuals with asthma and COPD (population) in managing their conditions in the community? (context)
2. What are the patient outcomes and experience of using CAs?

### Stage 2: Identifying Relevant Studies

A systematic search was conducted across 9 databases (CINAHL, CENTRAL, Embase, IEEE Xplore, PubMed, ProQuest Theses & Dissertations, Scopus, Web of Science, and Google Scholar) for articles published from January 1, 2014, to February 22, 2025. Reference lists of relevant reviews and included studies were also manually screened. For Google Scholar, the first 200 records ranked by relevance were screened, a pragmatic approach commonly applied in gray-literature searching [25], and consistent with recommendations to focus on the first 200 to 300 Google Scholar results when the platform is used to supplement gray-literature searching [26]. ProQuest Theses & Dissertations was searched in full as the principal gray-literature source. References published after the search closing date are cited in the Introduction and Discussion sections for background and contextual purposes only. They were not included in screening, eligibility assessment, data extraction, or synthesis.

The full search strategy was developed using the Peer Review of Electronic Search Strategies (PRESS) checklist [27] and refined in consultation with a research librarian. Truncations (*) and Boolean operators (AND, OR) were used to maximize search results. Keywords and MeSH terms such as “chatbots,” “conversational agents” were truncated and exploded. Search strategies for each database are presented in Multimedia Appendix 2.

### Stage 3: Study Selection

#### Population

Patients diagnosed with asthma or COPD were included, as these are the 2 most common lung diseases [1]. Both share similarities in clinical presentation and management needs, making them appropriate for combined analysis. Caregivers of individuals with asthma or COPD were included, given their crucial role in managing and supporting both pediatric and geriatric populations [28]. Other disease types and obstructive respiratory disease subtypes, such as cystic fibrosis, bronchiolitis, and bronchiectasis, were excluded due to their lower prevalence and distinct management requirements.

#### Concept

For inclusion, a CA was defined as a system that exchanges information with a user through natural language (written or spoken) in a turn-taking exchange and that generates its responses independently of human intervention. Eligible systems could be stand-alone software or an integrated chat feature within mobile apps. Examples include, but were not limited to, rule-based chatbots, text-based chatbots, voice-based agents, LLM-based chatbots, and embodied CAs. To be eligible, the agent had to be developed, adapted, or applied specifically to asthma or COPD care and to be intended for use by patients or their caregivers rather than by health care professionals for clinical decision-making. A total of 2 included reports [29,30] evaluated LLM output on asthma-specific questions but involved no conversational interaction with patients and were therefore reported separately and excluded from the synthesis of roles and patient outcomes. Conversational interfaces used for decision-making by health care professionals were excluded, as the focus is on patient-facing technologies.

#### Context

Only studies using CAs in home or community settings, regardless of country, were considered. Studies conducted in hospitals or residential care settings were excluded.

#### Type of Study

To synthesize a comprehensive range of evidence, no restrictions were placed on study design. Both empirical primary studies and gray literature were included. Reviews, websites, blogs, newspaper articles, and studies without full texts (despite attempts to contact the author) were excluded. Only English-language studies published from 2014 onwards were considered, as the use of CAs became popular in the mid-2010s, after the release of voice-based assistants [13].

#### Screening Process

All citations were imported into Rayyan reference manager, where duplicates were removed. The remaining citations were uploaded to Rayyan software for the screening process. Two reviewers (NBH and SQY) independently screened the articles, with disagreements resolved through discussion; a third reviewer (YJ) was available to arbitrate unresolved disagreements.

### Stage 4: Charting the Data

The first author (NBH) used the Joanna Briggs Institute (JBI) Data Extraction form to extract key information, including authors, year of publication, publication status, study location, intervention duration, study populations, methodology, key results, and features of CAs. A second reviewer (SQY) independently extracted data from a random sample of 20% reports (4/16) for verification as a pragmatic compromise given resource constraints, and the remaining extractions were checked against the source articles by the coauthors, with discrepancies resolved through discussion.

Where multiple reports originated from the same underlying study, the study was treated as the unit of analysis for participants, design, and setting. Reports were counted separately only for publication counts. Sloots et al [31] and ter Stal et al [32] report on the same cohort of 11 participants, and these participants are counted once. In the narrative synthesis, findings from these 2 reports were treated as one evidentiary contribution rather than independent corroboration.

### Stage 5: Collating, Summarizing, and Reporting the Results

Consistent with scoping review methodology, findings were mapped descriptively across study designs and stages of development rather than pooled, as the aim was to characterize the breadth of an emerging evidence base. An inductive content analysis approach [33] was used to synthesize the findings. For quantitative studies, relevant results sections were analyzed. For qualitative or mixed method studies, analysis included authors’ interpretations, participant quotations, and the interpretation of qualitative findings in the discussion. A 4-step process (decontextualization, recontextualization, categorization, and compilation) was used to synthesize and organize findings into categories and subcategories, which a second reviewer (SQY) subsequently reviewed. Tables and figures were created to support and illustrate findings. As a scoping review aims to outline existing literature, no quality appraisal of studies was conducted. However, to provide a descriptive overview of the evidence base, we recorded each study’s design, sample size, comparator presence and type, exposure duration, and use of clinically validated outcome instruments.

The Patterns, Advances, Gaps, Evidence for Practice, and Research Recommendations (PAGER) framework guided the discussion and critique of the included studies [34]. We adopted these reflective questions:

- Patterns: What patterns or groupings emerge from the analysis? What patterns can be observed within and across different groupings and categories?
- Advances: What discoveries or advances were made in the included studies? What requires further elaboration?
- Gaps: What questions remain unanswered in previous research? Which areas warrant further research, and how can they be prioritized? What has been thoroughly investigated such that further inquiry is unnecessary?
- Evidence for practice: What main points must be conveyed to stakeholders? What are the practical implications?
- Research recommendations: How can the review findings inform future research? What does not require additional research?

## Results

### Search Results

The search yielded 6275 citations, from which 2204 duplicates were removed. Following title and abstract screening, 27 articles were retrieved for full-text assessment and 16 reports from 15 studies [29-32,35-46] were included in this review (Figure 1).

**Figure 1.**
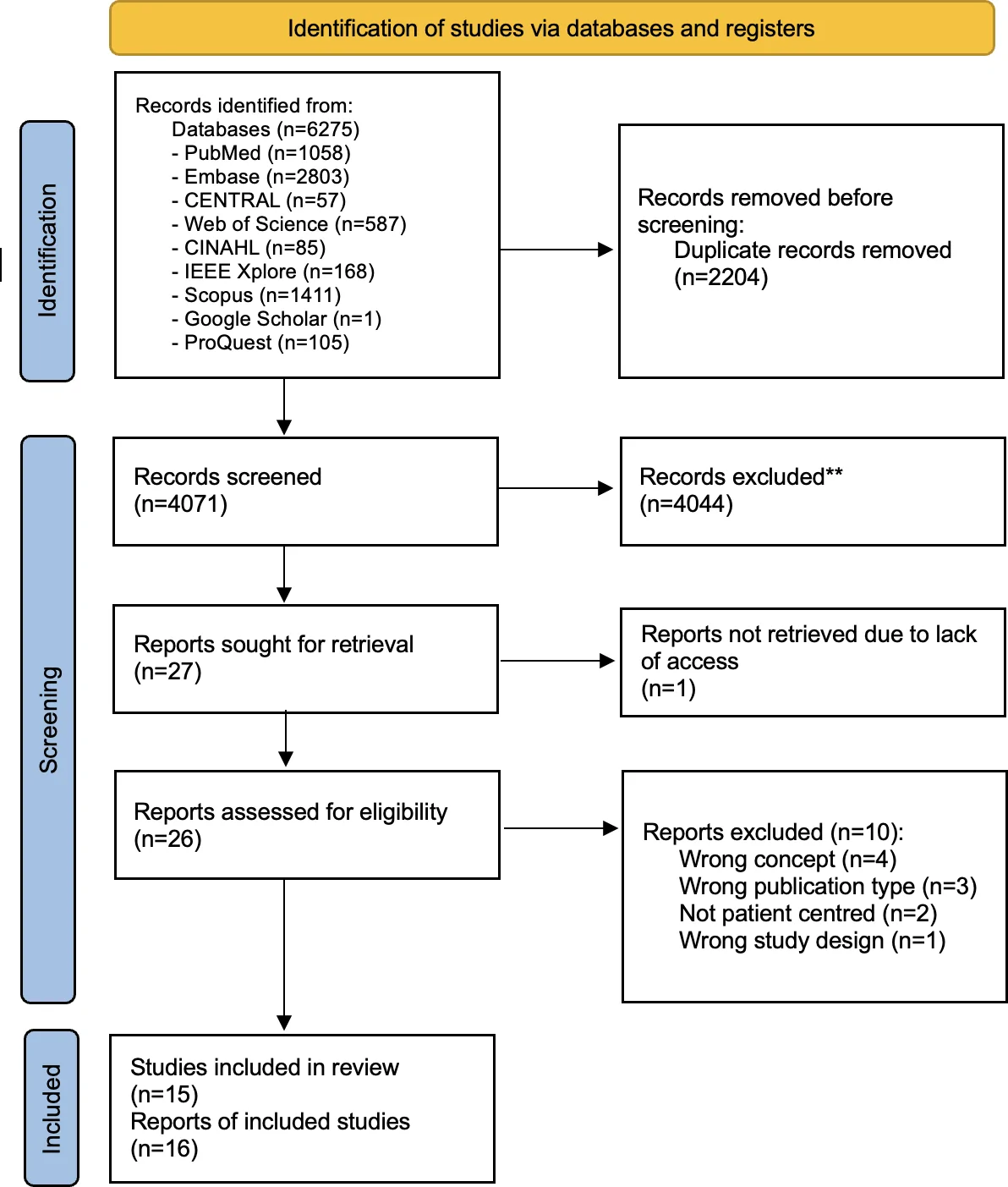
PRISMA (Preferred Reporting Items for Systematic Reviews and Meta-Analyses) diagram.

### Characteristics of Studies

Table 1 presents the characteristics of the included studies. The studies spanned 9 countries, including Switzerland (n=4) [35,39,41,42], the United Kingdom (n=3) [36,37,43], the United States (n=2) [40,44], the Netherlands (n=2; Sloots et al [31] and ter Stal et al [32], which report the same study), Denmark (n=1) [30], Saudi Arabia (n=1) [29], France (n=1) [38], Portugal (n=1) [45], and one additional study [46] conducted jointly in Denmark and Norway. A total of 3 [32,40,45] reports were conference papers and the remainder peer-reviewed journal articles.

The study designs varied, including cross-sectional questionnaire (n=1), mixed methods (n=3), qualitative (n=2), quantitative (n=2), randomized controlled trial (n=1), single-arm quasi-experimental (n=1), and single-arm quasi-experimental collecting qualitative and quantitative data (n=6), summing to the 16 reports. Most studies were pilot feasibility or developmental studies, reflecting the nascency of CA application in chronic respiratory disorders.

A total of 860 participants took part across the included reports. This included people with asthma or COPD, caregivers, and parent-child dyads, health care professionals, and researchers. Kadariya et al [40] tested kBot with 8 asthma clinicians and 8 researchers, Easton et al [37] included 5 health care professionals among 23 participants, Høj et al [30] involved 5 medical professionals only, and Quinde et al [43] included 2 experts among 10 participants. Alabdulmohsen et al [29] did not involve human participants, instead evaluating 30 questions posed to ChatGPT (OpenAI). Sloots et al [31] and ter Stal et al [32] report the same 11 participants, who were counted once. Participant numbers were largely driven by 3 studies: Gross et al [39] (n=240), Gonsard et al [38] (n=194), and Cook et al [36] (n=150).

**Table 1.** Characteristics of included studies (16 reports from 15 studies)^a^.

Author, year and country	Study design and type of publication	Aim of study	Disease	Population tested on			Description of intervention Use of CA^b^
			Eligibility criteria	Sample size	Age (years)	Duration	
Alabdulmohsen et al [29], 2024 Saudi Arabia	Quantitative Peer-reviewed journal article	Evaluate ability of ChatGPT to answer frequently asked questions about asthma with consistent reliability, acceptability, and easy readability	Asthma	NA^c^ but 30 asthma-related questions were asked	NA	NA	30 frequently asked questions of asthma were asked independently in the ChatGPT (OpenAI) App in 2 different devices in 2 different locations. The chat box's initial response to the questions without regeneration was collected and compared for consistency and reproducibility.
Cleres et al [35], 2021 Switzerland	Single-arm quasiexperimental: both qualitative and quantitative data collected (but no mention of mixed method) Conference paper	Investigate acceptability of a voice-based approach Lena to alleviate patients’ communication burden while filling questionnaires, the conversational agent’s ability to formulate its questions simply and understandability, and ability to understand the patient’s answers	COPD^d^	N=4 (male: 3, female: 1)	Mean (SD): 69 (5)	1 session	Lena will ask questions aimed at evaluating the patient’s health status, and a question referring to the patient’s mood. It would react differently based on the patient’s answer to the last question.
Cook et al [36], 2024 United Kingdom	Mixed methods Peer-reviewed journal article	Evaluate Brisa, a chatbot designed to improve self-assessment and self-management in patients with asthma	Asthma	N=150	Mean (SD): 39.36 (12.57)	28 days	Users can use the chatbot to measure the risk of an asthma attack, track their asthma control weekly, answer their questions on asthma management, help users identify triggers that exacerbate their symptoms, and provide strategies to help users manage their triggers.
Easton et al [37], 2019 United Kingdom	Qualitative Peer-reviewed journal article	To co-design the content, functionality, and interface modalities of an autonomous virtual agent to support self-management for patients with an exemplar long-term condition (COPD) and then to assess the acceptability and system content	COPD	N=23 Workshop phase: patients with COPD (n=6) and health care professionals (N=5) Scenario testing: patients with COPD (N=12)	Workshop 1, median (IQR): 82.5 (69-86) Workshop 2, median (IQR): 73 (66-80) Scenario testing, median (IQR): 71 (56-86)	1 session	The study developed and tested an AI-powered chat assistant designed to support individuals with both physical and mental health conditions by providing personalized information, self-management guidance, and emotional support through interactive conversations.
Gonsard et al [38], 2023 France	Mixed methods Peer-reviewed journal article	To assess whether children and adolescents with asthma would be ready to use a digital twin system	Asthma	N=194 Parent-child dyads (99 children and 95 parents) Children (male: 65, female: 34)	Mean (SD): 12.3 (2.8)	1 session	The study explored children’s perspectives on using AI-powered chat agents and digital twin technology to support their daily asthma management, and gauge their preference as compared to their current treatment.
Gross et al [39], 2021 Switzerland	Cross-sectional questionnaire Peer-reviewed journal article	To investigate preferences of patients with COPD for conversational agent-delivered interaction styles	COPD	N=240 Study 1=117 (male: 66, female: 51) Study 2=123 (male: 76, female: 47)	Study 1, mean (SD): 65.67 (10.92) Study 2, mean (SD): 67.82 (9.37)	3 months	AI chatbots’ communication styles were personalized to match the patient’s preferences to interact with chronic disease patients.
Høj et al [30], 2024 Denmark	Quantitative Peer-reviewed journal article	To evaluate scientific reliability of ChatGPT as a source of information on asthma	Asthma	Medical professionals specialized in allergic and respiratory diseases (n=5)	NA	NA	The study assessed ChatGPT’s reliability as a chat-based AI agent for providing information about asthma. Medical professionals evaluated the accuracy and quality of its responses to asthma-related questions.
Kadariya et al [40], 2019 United States	Single-arm quasiexperimental: Both qualitative and quantitative data collected (but no mention of mixed methods) Conference paper	Preliminary evaluation of kBot, a knowledge-enabled personalized chatbot system designed for health applications and adapted to help pediatric patients with asthma (age 8-15 years) to better control asthma	Asthma	N=16 8 asthma clinicians 8 researchers	NR Target population: 8-15	NA	kBot helps patients with asthma manage their condition. kBot interacts with users to provide tailored advice, reminders, and education based on each patient’s own health data and asthma management guidelines, supporting better self-care and symptom monitoring.
Kohlbrenner et al [41], 2024 Switzerland	Randomized controlled trial Peer-reviewed journal article	To investigate the effectiveness of a 12-week hybrid virtual coaching solution on health-related quality of life, with telemonitoring of clinical parameters delivered through the CAir Desk (consisting of smartphone, wrist-worn physical activity monitor, and environmental air quality monitor) in patients with stable COPD	COPD	N=30 (male: 17, female: 13) Intervention=21 (male: 12, female: 9) Control (telemonitoring only)=9 (male: 5, female: 4)	Mean (SD): 63 (9)	12 weeks	A hybrid model combines virtual coaching and telemonitoring to support patients with COPD. Patients receive personalized digital coaching, health education, and reminders, while their health data is remotely monitored, enabling tailored feedback and improved disease management.
Kowatsch et al [42], 2021 Switzerland	Single-arm quasiexperimental: both qualitative and quantitative data collected (but no mention of mixed methods) Peer-reviewed journal article	(1) Describe the design of MAX, a CA-delivered asthma intervention that supports health care professionals targeting child-parent teams in their everyday lives (2) To assess the reach of MAX, conversational agent-patient working alliance, acceptance, intervention completion rate, cognitive and behavioral outcomes, human effort, and responsiveness of health care professionals in primary and secondary care settings	Asthma	N=49 patients (male: 33, female: 16) N=48 supporting family members	Mean (SD) 12.04 (1.54)	4 weeks	CAs facilitate communication and coordination among health care professionals, patients, and family members in chronic disease management. These agents support self-care, information sharing, and collaborative decision-making for more effective asthma management.
Quinde et al [43], 2020 United Kingdom	Single-arm quasiexperimental: both qualitative and quantitative data collected (but no mention of mixed methods) Peer-reviewed journal article	To monitor their asthma health status	Asthma	N=10 (6 asthma, 2 experts, 1 nonprofessional carer, and 1 person with asthma and nonprofessional carer	NR^e^	50- to 80-minute demonstration	ADAPT integrates real-time contextual data with individual patient information, delivering tailored guidance and support to help patients manage their asthma more effectively.
Rhee et al [44], 2014 United States	Single-arm quasiexperimental: Both qualitative and quantitative data collected (but no mention of mixed methods) Peer-reviewed journal article	To develop and evaluate the feasibility and acceptability of a comprehensive mobile-phone-based asthma self-management aid for adolescents designed to facilitate symptom monitoring, treatment adherence, and adolescent-parent partnership	Asthma	N=32; 16 adolescent-parent dyads (male: 9, female: 6 as 1 dyad was excluded due to unsuccessful attempt to initiate the trial)	Mean (SD): 15 (1.5)	2 weeks	The app targeted to young users provides personalized reminders, symptom tracking, and educational resources to support effective asthma self-care. It uses features such as a user-friendly design, games, and age-appropriate educational content to appeal to young users.
Rodrigues et al [45], 2022 Portugal	Single-arm quasiexperimental: Both qualitative and quantitative data collected (but no mention of mixed methods) Conference paper	To develop and evaluate a conversational agent	COPD	N=15	67% >40 years old	NR	CA promotes physical activity among patients with COPD. It provides personalized motivational messages, activity tracking, and tailored feedback to support patients in increasing and maintaining their physical activity levels.
Sloots et al [31], 2021 the Netherlands	Single-arm quasiexperimental Peer-reviewed journal article	Same study as ter Stal et al, 2021 [32]	COPD and heart failure	N=11 (male: 7, female: 4)	Mean (SD): 66.8 (2.9)	4 months	Part of eHealth self-management intervention (training sessions, monitoring, inhaler, information, exercise and physical activity modules) CA gave reminders on completing to-do list, feedback on inhaled medication adherence and technique, and recommendations to promote healthy behavior.
ter Stal et al [32], 2021 the Netherlands	Mixed methods Peer-reviewed journal article	To investigate how patients perceive the design of the embodied conversational agent over time with regard to the embodied CA characteristics (friendliness, trustworthiness, involvement, expertise, and authority), small talk interaction, and likeliness of following the agent’s advice (same study as ter Stal Sloots et al, 2021 [32]).	COPD and heart failure	N=11 (male: 7, female: 4)	70 (median)	4 months	Same as Sloots et al, 2021 [31]
Wegener et al [46], 2024 Denmark and Norway	Qualitative Peer-reviewed journal article	To describe the perspective, needs, and preferences of older adults living with COPD in the context of the design and development of a conversational agent	COPD	N=33	NR	2-hour cocreation workshop	First phase of workshop: elaborate on early ideas and narrow down to a few prioritized functions. Second phase: present conversational agent work-up and gather input that could be used to develop conversational agent prototype version 1.

a Sloots et al (2021) [31] and ter Stal et al (2021) [32] report the same 11-participant cohort and constitute one study; those participants are counted once. Alabdulmohsen et al (2024) [29] and Høj et al (2024) [30] evaluate large language model output against fixed question sets and involve no conversational interaction and no patient use; they are reported separately and are excluded from the synthesis of roles and patient outcomes.

b CA: conversational agent.

c NA: not applicable.

d COPD: chronic obstructive pulmonary disease.

e NR: not reported.

### Results of Synthesis

The findings were divided into 4 categories: (1) roles of CAs, (2) direct patient outcomes, (3) indirect patient outcomes, and (4) evaluation of the LLM output. The summary of synthesis findings is presented in Table 2 (detailed synthesis findings can be found in (Multimedia Appendix 3).

**Table 2.** Summary of synthesis findings. Roles and outcomes are as reported by the source studies. Sloots et al (2021) [31] and ter Stal et al [32] (2021) report the same study.

Type of CA^a^	Defining feature	Studies in this review	Roles	Outcomes
Text-based	Written turn-taking exchange without avatar or speech interface	Rhee et al [44] 2014 Easton et al [37] 2019 Gross et al [39] 2021 Cook et al 2024 [36] Kadariya et al [40] 2019	Education on medication use, triggers, and management strategies Symptom and control tracking; reminders Risk assessment Motivational and emotional support Generation of reports for discussion with clinicians	Improved self-management and medication adherence, self-reported [44]. 8.38% improvement in Asthma Control Questionnaire after 28 days, with trigger-avoidance intentions from 30.8% to 66.7% [36] system usability median 73.75 [37]. Usability 83.1 for clinicians and 82.8 for researchers, with no patient users [40]. Working alliance and intention to continue varied by age, COPD^b^ severity, and disease literacy, n=240 [39].
Voice-based agent	Speech input and output; hands-free interaction	Cleres et al [35] 2021 Quinde et al [43] 2020 Rodrigues et al [45] 2022	Daily assessment of health status and mood Asthma control assessment Contextual monitoring Medication reminders Physical activity promotion with tailored feedback	Perceived ease of use, enjoyment, usefulness, and intention to use rated agree to strongly agree, n=4 [35]. Virtual assistant rated useful to very useful but the least important system feature, n=10 [43]. Tasks completed successfully by voice, with recognition failures for particular words, accents, and dialects reported across all three [35,43,45].
Embodied conversational agent	Visual avatar accompanying dialogue	Sloots et al [31] 2021 ter Stal et al [32] (one study) 2021	Reminders on daily tasks Feedback on inhaled medication adherence and technique Recommendations to promote healthy behavior	High diary completion and high inhaler adherence but poorly performed inhalation technique, with self-efficacy and quality of life unchanged and no statistical testing reported [31]. Participants less likely to follow agent advice (*P*=.01), with advice perceived as poorly fitted to individual circumstances and mixed views on cartoon appearance [32].
Virtual coach with telemonitoring	Structured program delivered conversationally alongside remote monitoring and clinician involvement	Kowatsch et al [42] 2021 Kohlbrenner et al [41], 2024	Structured education via quizzes and video Review of inhalation technique by clinicians Coaching on daily living and physical activity Sensor-based monitoring	Significant increase in asthma knowledge, with 49.5% of young patients installing and starting to use the agent [42]. Randomized evidence in this review (n=30), with quality of life, symptom burden, and exercise capacity improving in both arms and no statistically significant between-group differences [41].
Concept or prototype system assessed through user perspectives	Participants responded to described or co-designed systems rather than using a deployed agent	Gonsard et al [38] 2023 Wegener et al [46] 2024	Anticipated roles Remote and real-time asthma management Education on lung capacity Sleep support Optional data sharing with clinicians and caregivers	Willingness to use: 56% if equivalent to physician care, 93% if 30% more effective, and 23% preferring the system even when 10% less effective [38]. Concerns about loss of humanity, 24%, and effects on autonomy [38]. Unwillingness to replace clinician contact and mixed views on monitoring frequency [46].
LLM^c^ output evaluation	Model evaluation with a fixed question set and rated by experts; no deployed agent	Alabdulmohsen et al [29] 2024 Høj et al [30] 2024	None Information quality only	93.3% of responses rated good reliability and 78.3% acceptable as the sole answer for a patient, with 2 of 30 questions answered inconsistently [29]. 81% scored 4 of 5 or higher for accuracy [30] readability rated difficult in both [29,30].

a CA: conversational agent.

b COPD: chronic obstructive pulmonary disease.

c LLM: large language model.

### Roles of CAs

CAs supported patient needs across 3 key roles. First, they facilitate information dissemination and patient education [30-32,36-39,42,46]. Some provided on-demand responses to patient queries, while others delivered structured, tailored educational content to improve disease understanding and self-management [41,42]. Second, several agents monitor patient data, including physiological parameters, self-reported health status, and validated patient-reported outcome measures. Third, CAs assumed a coaching role by helping patients identify potential triggers, estimating exacerbation risk, and delivering timely reminders for medication adherence and physical activity [31,32,35-38,41-44,46]. Some CAs extend their coaching capabilities to emotional and psychosocial domains. By offering motivation, companionship, and empathy-driven dialog, they empower patients to maintain positive self-management behaviors and reduce the psychological burden of chronic disease [36,42,44].

### Direct Patient Outcomes

Direct patient outcomes are quantifiable indicators of disease management (behavioral changes) and disease progression (clinical outcomes). A total of 6 reports described behavioral outcomes, such as lifestyle modifications and increased self-management [31,36,39,41,42,44]. Reported changes included improved inhaler technique [42], increased physical activity, and improved adherence to prescribed medications and treatment plans [44]. Participants in one study also reported greater empowerment and proactivity in managing their conditions [44]. All of these observations arise from single-arm or uncontrolled designs. Sloots et al [31] observed partial engagement, in which participants initiated components such as maintaining daily diaries but did not follow through with key recommended actions, including contacting case managers or demonstrating correct inhaler technique. The companion report by ter Stal et al [32], analyzing the same 11-participant cohort, described deficiencies in character design and responsiveness.

Only 4 studies measured clinical outcomes, including asthma control, symptom burden, functional exercise capacity, and lung function [31,36,41,42]. Of these, one was a randomized controlled trial (n=30), in which 21 participants were allocated to the intervention and 9 to the control group [41]. The remaining 3 studies did not include a control or comparison group. The single statistically significant clinical result was an 8.38% improvement in Asthma Control Questionnaire score reported by Cook et al [36] over a 28-day single-arm study without a comparator. Sloots et al (2021) [31] reported mixed findings. Taken together, these findings suggested that the current evidence does not provide a robust basis for drawing conclusions regarding the effects of CAs on clinical outcomes in asthma or COPD.

### Indirect Patient Outcomes

Indirect patient outcomes capture the practical aspects of CAs’ usage, including user satisfaction and engagement. Usability and feasibility were the most frequently reported outcomes across the reviewed studies [31,32,35-46]. Two studies [40,44] reported that participants described CAs as convenient, easy to use, and helpful in delivering accessible health information, and that participants valued immediate responses and interactive, personalized communication.

Other studies [32,36,45] reported substantial usability problems, including poor conversational flow, limited adaptability, and instances in which the CA failed to understand user inputs or intent, which participants linked to frustration and reduced engagement.

Reports on the trustworthiness and credibility of CAs varied. Some participants described reluctance to follow CA-generated advice without consulting a health care professional [32], and some were hesitant to recommend the agent to others or rely on it for decisions about their care [38]. Other participants described the advice as helpful and were open to incorporating the agent into their self-management routines [36].

Participants expressed preferences regarding agent functionality and design, including gender, appearance, interaction style, and communication tone [32,36,37,39,43,45]. Visual design was reported to influence user acceptance, with participants in 1 study [32] favoring realistic representations such as a professionally dressed nurse over cartoon-like figures, which were described as more credible and authoritative. Reported gender preference favored female agents [32] or agents matching the participants’ own gender [42]. In terms of communication style, participants preferred a casual, empathetic tone over formal or clinical language [45].

From a functionality perspective, users valued tailored support features, including text-based interaction [32,37], multilingual support [35,45], and disease-tracking and management features [37,43]. These perspectives often varied by patient characteristics, including age, COPD severity, and recency of diagnosis [35,45], highlighting the importance of aligning CAs’ design with user demographics, communication preferences, and perceived authenticity to enhance engagement and trust.

### Evaluation of LLM Output

A total of 2 [29,30] reports evaluated LLM output for respiratory disease care. Alabdulmohsen et al [29] posed 30 frequently asked asthma questions to ChatGPT across 2 devices in 2 locations and reported consistent and reproducible responses, while noting that readability exceeded recommended levels for patient-facing materials. Høj et al [30] had 5 specialists in allergic and respiratory disease rate ChatGPT responses on asthma and reported generally accurate content. These studies therefore provide evidence on the accuracy, consistency, and readability of LLM-generated asthma information under controlled test conditions, but they do not examine conversational interaction, patient use, trust, engagement, or support for self-management in community settings. For this reason, their findings are treated separately from studies of patient-facing CAs and were not used to support claims about patient experience, agent role, or implementation in community-based condition management.

## Discussion

This scoping review synthesizes current evidence on the use of CAs in the management of asthma and COPD. The findings highlight that the current evidence remains limited. Among the included studies, most were small feasibility, developmental, or pilot studies. Only 4 [31,36,41,42] measured clinical outcomes, and just one randomized controlled trial was identified. The literature provides a descriptive map of the roles CAs have been designed to perform, the outcomes studied and less explored, and the recurring user-experience issues that future implementation must address. Table 3 presents a detailed critique using the PAGER framework.

The limited strength of evidence is not unique to respiratory care. Reviews of AI in mental health report a similar evidence pattern in existing literature: rapidly expanding applications, recurring concerns about limited dataset diversity, algorithmic bias, and insufficient clinical validation [47]. Similar gaps between optimism and proven outcomes have also been reported for CAs in noncommunicable diseases more broadly [48,49]. Therefore, the limited evidence found in this review may reflect a field-wide stage of development, rather than a problem specific to respiratory applications.

**Table 3.** Patterns, Advances, Gaps, Evidence for Practice, and Research Recommendations framework.

Pattern	Advances	Gaps	Evidence for practice	Research recommendations
Characteristics of studies	Varied study designs were used to evaluate the CAs^a^, including qualitative methods, which is encouraging. Most were conducted in Western societies.	Research in Eastern societies is lacking. Only one randomized controlled trial has been published. More good quality, in-depth evaluative studies are needed, such as randomized controlled trials and mixed method studies.	Due to the early stage of development in this field, CAs are not recommended for use in clinical practice yet.	More studies should be conducted in Eastern societies. More randomized controlled trials and in-depth evaluative studies are needed. There were studies using both qualitative and quantitative data, but without using an established methodology such as mixed-methods, or multimethods to properly integrate both forms of data in the synthesis. Future studies should adhere to reporting guidelines and methodological guidance to ensure good quality research.
Roles of CAs	CAs have roles that are consistent with their applications in other diseases	While most functioned as needed to provide information, it was only in two studies that CAs acted in the form of a structured educational program. It will be of interest to know if this is a desired and effective feature in this patient population in future iterations of CAs evaluated.	The roles of CAs are unsurprising considering the broader literature of CAs in disease management and monitoring, but the amount and quality of evidence in this population is weak, as seen in Table 2.	More in-depth evaluative studies are needed in terms of how effective these roles are performed quantitatively and qualitatively, and whether they translate into better clinical outcomes.
Direct patient outcomes	Some studies have investigated the impact of CAs on clinical and/or behavioral changes. Some interesting findings include how the type of conversational style impacts behavioral changes which warrants further research, and how potential deficiencies in character design caused users to be less likely to follow the CA’s advice.	Only one randomized controlled trial has been published. More good quality, in-depth evaluative studies are needed. Character design and its suitability for different patient groups may need to be optimized and it is important that this be evaluated in the context of direct patient outcomes.	Due to the early stage of development in this field, CAs are not recommended for use in clinical practice yet.	More randomized controlled trials and in-depth evaluative studies are needed. Clinical outcomes must be investigated in future research to determine the benefits or harms of CAs in disease management.
Indirect patient outcomes	Patients’ experiences and specific preferences were explored, though the findings were quite heterogeneous. Technical issues and limitations in conversational capabilities were present in some studies.	Perceptions of CAs have been thoroughly investigated. More good quality, in-depth evaluative studies are needed.	Due to the early stage of development in this field, CAs are not recommended for use in clinical practice yet.	This is a commonly investigated finding and is easily obtained from participants, but future studies should adhere to reporting guidelines and methodological guidance to ensure good quality research.

a CA: conversational agent.

### Roles of CAs

The systems described in the reviewed studies extend beyond traditional symptom monitoring and tracking to include emotional and psychosocial support [35,37] and the promotion of physical activity [31,32,45]. This shift suggests that developers are increasingly treating psychosocial and behavioral domains as part of chronic respiratory care alongside disease monitoring, reflecting a more holistic and preventive care approach.

Managing chronic conditions is often stressful and associated with poorer mental health [50]. CAs have been proposed as virtual companions offering support, encouragement, and reassurance, with anonymity providing a nonjudgmental avenue for users to express concerns. Prior research has investigated CAs for mental health support [47,51], and similar approaches are being adapted for chronic respiratory disease management. However, in the current review, none of the included studies measured psychological outcomes using validated instruments, so this role is described but not evaluated. Questions also remain about the authenticity of such interactions and their capacity to establish a meaningful therapeutic relationship.

### Direct Patient Outcomes

Despite growing interest in the use of CAs in health care, few studies have evaluated their impact on direct patient outcomes in asthma and COPD management, and those that have done so were not designed to support causal inference. It therefore remains premature to conclude whether these technologies lead to measurable improvements in clinical indicators. This observation aligns with previous reviews, which identified the scarcity of studies assessing the clinical impact of CAs and the absence of rigorous randomized controlled trials supporting effectiveness in disease management [48,52]. One plausible explanation is that much of the existing literature is pilot or developmental in nature, prioritizing feasibility, usability, and patient perceptions over clinical endpoints. Another possible explanation could be that the short exposure durations typical of this literature may not be sufficient to detect changes in outcomes such as exacerbation frequency or lung function. Both reflect the early and exploratory phase of integrating CAs into chronic disease care [52].

### Indirect Patient Outcomes

While several studies reported high usability and feasibility scores for CAs, these findings derive largely from small, self-selected samples in single sessions and may not reflect the needs of all users, particularly older adults with COPD. A systematic review of digital health adoption among older adults with chronic disease identifies limited digital literacy, physical and cognitive challenges, infrastructural deficits, usability problems, and mistrust as recurring barriers, with older women showing lower adoption [53]. A longitudinal pilot study of a COPD app for older adults found that technology-related barriers decreased with familiarity, whereas perceived lack of relevance and competing health concerns persisted [54]. Language complexity may pose further difficulty for users with lower health literacy, a concern reinforced by the readability problems documented in the LLM evaluations [29]. Health care providers should account for specific users’ literacy, digital competencies, and engagement when considering CAs [55].

Trust was a recurring concern, and reports of hesitancy were more common for text-based and embodied agents than for a single voice-based agent in this review. Trust in conversational systems is consistently associated with perceived social presence and with perceived warmth and competence, and is undermined by communication delays [56-58]. Voice interfaces can convey tone, timing, and natural turn-taking cues that text cannot, making them richer in these aspects. They may also reduce access barriers that are especially relevant for people with COPD, many of whom are older adults, by avoiding difficulties related to small text, typing accuracy, and visual acuity [53]. Nevertheless, this review included only one voice-based agent, evaluated in 4 participants [35]; therefore, future studies should further examine whether the text-versus-voice distinction remains relevant in chronic respiratory care.

Across the wider literature, many users may still see CAs as less trustworthy than clinicians for complex needs. Clearly communicating the limits of these systems, including that they are not substitutes for professional advice, is essential for setting realistic expectations. Across health care apps, a hybrid model has been proposed in which the agent acts as a first point of contact, with structured escalation to a health care professional when needed [59], and it has been evaluated in mental health, where an AI-delivered program with clinician oversight achieved outcomes comparable to face-to-face cognitive behavioral therapy, while reducing clinician time by up to eightfold [60]. Clinician endorsement and hybrid care models have also been identified as facilitators of digital health adoption among older adults with chronic disease [53]. No included study evaluated such a model in asthma or COPD, so future research should test whether this approach is feasible and effective in chronic respiratory care.

A notable descriptive finding is that users expressed specific preferences regarding CA’s characteristics, including gender, age, appearance, interaction styles, and communication tone. Consistent with prior work, younger and female agents were generally preferred over older and male agents [61]. Possible explanations include that female agents may be perceived as more empathetic or nurturing because of wider social stereotypes about gender [62]. Nevertheless, the preference findings in this review are based on only 2 reports [32,42], and neither study tested the reasons behind these preferences. These findings should therefore be treated as design signals for future testing, rather than as evidence of broader societal attitudes. Personalization may improve comfort and sustained use, but it should be implemented carefully to avoid reinforcing stereotypes or introducing design bias. An inclusive and authentic design approach may help improve both user experience and ethical practice.

### LLMs

As patients may increasingly use LLMs for treatment information or self-management advice, a parallel body of work has evaluated the accuracy of LLMs in answering standardized respiratory questions after the search closing date in this review [15-17,63,64]. Like the 2 LLM evaluations included in our review, they assess model output under test conditions rather than patient interaction, intervention delivery, or self-management support. Collectively, they indicated that LLM responses are often accurate, but readability, understandability, and performance on more complex scenario-based tasks remain concerns. Future work should examine how patients actually use LLMs for respiratory self-management and whether this use is safe, understandable, and clinically appropriate.

### Strengths and Limitations

A key strength of this review is its position as the first to map roles and outcomes of CAs in asthma and COPD management. Its methodological strengths include a broad search across 9 databases, the inclusion of gray literature, and the use of the PAGER framework.

However, several limitations should be noted. Restricting the review to English-language publications reduced cultural and linguistic diversity. Most included studies were conducted in Western countries; this pattern may partly reflect the English-language restriction, which could have led to the underrepresentation of research from Eastern and low- and middle-income countries. This limits the generalizability of the findings to Eastern and resource-limited contexts. Data were extracted by a single author (NBH), with verification by coauthors (SQY and YJ), rather than through full independent duplicate extraction. This approach may have introduced a risk of extraction error. Only one randomized controlled trial [41] with a small sample was identified, highlighting the lack of robust comparative evidence. Additionally, many studies were still in the formative phase, focused on development or preliminary testing. Consequently, further large-scale, rigorous research is needed before these findings can inform routine clinical practice or policy.

### Implications

This scoping review maps the roles that CAs have been designed to perform in asthma and COPD, namely patient education, symptom monitoring, and psychosocial support. Patients are the end users of these systems, and this review shows that problems at the point of interaction, including poor conversational flow, limited responsiveness, character design, and lack of personalization, were repeatedly associated with disengagement. In one project, these problems were also linked to noncompletion of the recommended self-management actions the agent was designed to prompt [31,32]. Patients should therefore be central to co-design from the outset, through sustained involvement across iterative development cycles rather than one-off consultation at the requirements stage. Several included studies did involve patients or caregivers in co-design [37,45,46], and these provided the most detailed and actionable insights into user requirements in this review.

Health care professionals also have an important role. The functions mapped in this review, including education, monitoring, and psychosocial support, align closely with nursing responsibilities in chronic respiratory care [65]. Nurses are therefore well placed to contribute to system design, guide patients in use, support digital literacy, and help interpret CA-generated information [66]. Doing so would require structured training and institutional support [67].

Implementation also depends on factors beyond the individual patient–agent interaction, many of which were largely absent from the included studies. These include reimbursement and procurement pathways, medical device and AI regulation, interoperability with electronic health records, digital infrastructure and connectivity, workforce training, and governance of patient data. Evidence from policy-driven digital health interventions suggests that these structural and policy conditions often determine whether clinical and system-level benefits are realized, regardless of the tool itself [68].

Privacy and data security require particular attention. Information collected by CAs must comply with relevant privacy regulations and ethical standards [69], and weaknesses in technical safeguards or the use of unregulated “shadow IT” may reduce acceptance, especially among younger users who are more skeptical of data harvesting [70]. Mixed user experiences and concerns about trust also point to the need for clinical oversight to ensure that CA-generated information is accurate and appropriate [71].

Nursing and clinical leadership will therefore be important in shaping the policies, governance arrangements, and training needed for safe and equitable use. Overall, the gap between the developmental studies reviewed here and real-world deployment remains substantial, and depends as much on policy and infrastructure as on the technology itself.

### Directions for Future Research

Most included studies were early-phase work, pilot, or developmental in design, limiting evidence to short-term feasibility outcomes and offering little insight into whether behavioral changes persist or translate into meaningful clinical improvements over time. Future research should prioritize longitudinal, rigorously designed trials to evaluate the sustained effectiveness of CAs in chronic respiratory care. Specifically, randomized controlled trials with adequate sample sizes, active comparators, and clinically meaningful endpoints are urgently needed. Adopting mixed methods frameworks in future work would further enable the integration of quantitative effectiveness data with qualitative insights into the patient experience, producing a more complete and actionable evidence base.

There is a pressing need to incorporate direct clinical outcome measures into CA research on chronic respiratory disease management. In the absence of evidence that CAs improve disease control, reduce exacerbations, or enhance medication adherence, their routine clinical use cannot be recommended. Future research should therefore prioritize validated disease-specific endpoints such as the Asthma Control Test (ACT), the COPD Assessment Test (CAT), or spirometric measures, and should report sample size calculations against those endpoints. To enable cross-study synthesis and meta-analysis, future work should align with established frameworks such as the Core Outcome Measures in Effectiveness Trials (COMET) initiative [72] to define and adopt a minimum core outcomes set for CA research in chronic respiratory disease.

A critical gap identified in this review is the near-total absence of research from low- and middle-income countries, despite their disproportionate burden of obstructive lung disease and rapidly growing interest in digital health solutions [1]. As noted in the limitations, our English-language restriction may have contributed to this gap. Future work should assess the feasibility, safety, and acceptability of CAs across diverse socioeconomic and health system contexts, with explicit attention to infrastructure, language, health literacy, and cultural norms. Older adults, who represent a large proportion of the COPD population and who demonstrated more variable and cautious attitudes toward CA use in this review, also remained understudied [53].

Co-design should place patients first. Given the technical and clinical complexity of CA implementation, co-design with end users, namely patients and caregivers, alongside health care professionals, including nurses, should be embedded in the development cycle from the outset rather than pursued retrospectively. This review found that deficiencies in character design, conversational flow, and responsiveness were frequently linked to reduced engagement and less favorable patient outcomes, underscoring the value of iterative, user-centered design processes. Future studies should document and report their co-design methodology transparently, including who was involved, at which stages, and how their input changed the resulting system, allowing replication and knowledge transfer across settings.

### Conclusion

In summary, this review mapped the use of CAs in the management of asthma and COPD and found that the current evidence base remains too limited to support conclusions about clinical benefit. Across the 15 included studies [29-32,35-46], the evidence base was mainly small feasibility, developmental, and pilot studies. Descriptively, CAs have been developed to support education, health data collection, and psychosocial support, while user-experience factors such as conversational flow, responsiveness, trust, and personalization consistently emerged as important for engagement. Future studies should be adequately powered, longitudinal, and use clinically meaningful, standardized outcomes across diverse settings, with patient input and clinical oversight. Until then, CAs in asthma and COPD remain a promising research area rather than tools ready for routine care. AI should complement, not replace, human relationships in chronic respiratory care.

## Supplementary material

10.2196/98338 Multimedia Appendix 1 Types of CA technology.

10.2196/98338 Multimedia Appendix 2 Search strategy.

10.2196/98338 Multimedia Appendix 3 Detailed synthesis findings.

10.2196/98338 Checklist 1 PRISMA-ScR checklist.

## Abbreviations

ACT: Asthma Control Test
CA: conversational agent
CAT: COPD Assessment Test
COMET: Core Outcome Measures in Effectiveness Trials
COPD: chronic obstructive pulmonary disease
JBI: Joanna Briggs Institute
LLM: large language model
mHealth: mobile health
NLP: natural language processing
PAGER: Patterns, Advances, Gaps, Evidence for Practice, and Research Recommendations
PRESS: Peer Review of Electronic Search Strategies
PRISMA-ScR: Preferred Reporting Items for Systematic Reviews and Meta-Analyses extension for Scoping Reviews

## References

[1] Halpin DMG, Masekela R, Vogelmeier CF (2026). Addressing the global challenges of COPD and asthma: a shared vision from the global initiative for chronic obstructive pulmonary disease (GOLD) and the global initiative for asthma (GINA). Arch Bronconeumol.

[2] Wang Z, Lin J, Liang L (2025). Global, regional, and national burden of chronic obstructive pulmonary disease and its attributable risk factors from 1990 to 2021: an analysis for the Global Burden of Disease Study 2021. Respir Res.

[3] Aldabayan YS (2023). Mental health and sleep quality among patients with asthma and COPD. Front Med.

[4] Eger K, Paroczai D, Bacon A (2022). The effect of the COVID-19 pandemic on severe asthma care in Europe: will care change for good?. ERJ Open Res.

[5] Al Mahmud A, Joachim S, Jayaraman PP (2026). Digital health interventions to support chronic disease management: systematic scoping review. JMIR Mhealth Uhealth.

[6] Zhuang M, Hassan II, W Ahmad WMA (2025). Effectiveness of digital health interventions for chronic obstructive pulmonary disease: systematic review and meta-analysis. J Med Internet Res.

[7] Meyerowitz-Katz G, Ravi S, Arnolda L, Feng X, Maberly G, Astell-Burt T (2020). Rates of attrition and dropout in app-based interventions for chronic disease. J Med Internet Res.

[8] Jiang Z, Huang X, Wang Z, Liu Y, Huang L, Luo X (2024). Embodied conversational agents for chronic diseases: scoping review. J Med Internet Res.

[9] Rowland SP, Fitzgerald JE, Holme T, Powell J, McGregor A (2020). What is the clinical value of mHealth for patients?. NPJ Digit Med.

[10] Beattie M, Zheng H, Nugent C, McCullagh P (2014). Self-management of COPD: a technology driven paradigm.

[11] Allouch M, Azaria A, Azoulay R (2021). Conversational agents: goals, technologies, vision and challenges. Sensors (Basel).

[12] Weizenbaum J (1966). ELIZA—a computer program for the study of natural language communication between man and machine. Commun ACM.

[13] Schöbel S, Schmitt A, Benner D, Saqr M, Janson A, Leimeister JM (2024). Charting the evolution and future of conversational agents: a research agenda along five waves and new frontiers. Inf Syst Front.

[14] Denecke K (2024). How do conversational agents in healthcare impact on patient agency?. https://aclanthology.org/2024.teicai-1.

[15] Yin Y, Zeng M, Wang H (2025). A clinician-based comparative study of large language models in answering medical questions: the case of asthma. Front Pediatr.

[16] Nigro M, Aliverti A, Angelucci A (2025). Artificial intelligence–generated answers to patients’ questions on asthma: the artificial intelligence responses on asthma study. J Allergy Clin Immunol Pract.

[17] Li PY, Gershon A, Kouri A (2026). Evaluating the accuracy of large language models in answering asthma multiple choice and objective structured clinical examination questions. Chest.

[18] Martins A, Londral A, L Nunes I, V Lapão L (2024). Unlocking human-like conversations: scoping review of automation techniques for personalized healthcare interventions using conversational agents. Int J Med Inform.

[19] Uetova E, Hederman L, Ross R, O’Sullivan D (2024). Exploring the characteristics of conversational agents in chronic disease management interventions: a scoping review. Digit Health.

[20] Laranjo L, Dunn AG, Tong HL (2018). Conversational agents in healthcare: a systematic review. J Am Med Inform Assoc.

[21] Montenegro JLZ, da Costa CA, da Rosa Righi R (2019). Survey of conversational agents in health. Expert Syst Appl.

[22] Arksey H, O’Malley L (2005). Scoping studies: towards a methodological framework. Int J Soc Res Methodol.

[23] Tricco AC, Lillie E, Zarin W (2018). PRISMA Extension for scoping reviews (PRISMA-ScR): checklist and explanation. Ann Intern Med.

[24] Hassan NB, Yoong SQ, Jiang Y (2025). Exploring the role of conversational agents in the management of obstructive lung diseases: A scoping review.

[25] Bramer WM, Rethlefsen ML, Kleijnen J, Franco OH (2017). Optimal database combinations for literature searches in systematic reviews: a prospective exploratory study. Syst Rev.

[26] Haddaway NR, Collins AM, Coughlin D, Kirk S (2015). The role of Google scholar in evidence reviews and its applicability to grey literature searching. PLoS One.

[27] McGowan J, Sampson M, Salzwedel DM, Cogo E, Foerster V, Lefebvre C (2016). PRESS Peer review of electronic search strategies: 2015 guideline statement. J Clin Epidemiol.

[28] Alsayed B, Alatawi A, Alatawi OM, Alatawi RH, Alfuhaymani AM, Aljohani JK (2023). Association of asthma control with caregivers’ knowledge and practices for children with asthma in the Tabuk Region of Saudi Arabia: a cross-sectional study. Cureus.

[29] Alabdulmohsen DM, Almahmudi MA, Alhashim JN (2024). Is ChatGPT a reliable source of patient information on asthma?. Cureus.

[30] Høj S, Thomsen SF, Ulrik CS, Meteran H, Sigsgaard T, Meteran H (2024). Evaluating the scientific reliability of ChatGPT as a source of information on asthma. J Allergy Clin Immunol Glob.

[31] Sloots J, Bakker M, van der Palen J (2021). Adherence to an eHealth self-management intervention for patients with both COPD and heart failure: results of a pilot study. Int J Chron Obstruct Pulmon Dis.

[32] Ter Stal S, Sloots J, Ramlal A, Op den Akker H, Lenferink A, Tabak M (2021). An embodied conversational agent in an eHealth self-management intervention for chronic obstructive pulmonary disease and chronic heart failure: exploratory study in a real-life setting. JMIR Hum Factors.

[33] Bengtsson M (2016). How to plan and perform a qualitative study using content analysis. NursingPlus Open.

[34] Bradbury-Jones C, Aveyard H, Herber OR, Isham L, Taylor J, O’Malley L (2022). Scoping reviews: the PAGER framework for improving the quality of reporting. Int J Soc Res Methodol.

[35] Cleres DM, Rassouli F, Brutsche M, Kowatsch T, Barata F (2021). Lena: a voice-based conversational agent for remote patient monitoring in chronic obstructive pulmonary disease.

[36] Cook D, Peters D, Moradbakhti L (2024). A text-based conversational agent for asthma support: mixed-methods feasibility study. Digit Health.

[37] Easton K, Potter S, Bec R (2019). A virtual agent to support individuals living with physical and mental comorbidities: co-design and acceptability testing. J Med Internet Res.

[38] Gonsard A, AbouTaam R, Prévost B (2023). Children’s views on artificial intelligence and digital twins for the daily management of their asthma: a mixed-method study. Eur J Pediatr.

[39] Gross C, Schachner T, Hasl A (2021). Personalization of conversational agent-patient interaction styles for chronic disease management: two consecutive cross-sectional questionnaire studies. J Med Internet Res.

[40] Kadariya D, Venkataramanan R, Yip HY, Kalra M, Thirunarayanan K, Sheth A (2019). KBot: knowledge-enabled personalized chatbot for asthma self-management.

[41] Kohlbrenner D, Kuhn M, Kläy A (2024). Hybrid virtual coaching and telemonitoring in COPD management: The Cair randomised controlled study. Int J Chron Obstruct Pulmon Dis.

[42] Kowatsch T, Schachner T, Harperink S (2021). Conversational agents as mediating social actors in chronic disease management involving health care professionals, patients, and family members: multisite single-arm feasibility study. J Med Internet Res.

[43] Quinde M, Augusto JC, Khan N, van Wyk A (2020). ADAPT: approach to develop context-aware solutions for personalised asthma management. J Biomed Inform.

[44] Rhee H, Allen J, Mammen J, Swift M (2014). Mobile phone-based asthma self-management aid for adolescents (mASMAA): a feasibility study. Patient Prefer Adherence.

[45] Rodrigues R, Caceiro R, Brites-Pereira M, Rocha A, Adeli H, Dzemyda G, Moreira F (2022). Information Systems and Technologies.

[46] Wegener EK, M Bergschöld J, Kramer T, Schmidt CW, Borgnakke K (2024). Co-designing a conversational agent with older adults with chronic obstructive pulmonary disease who age in place: qualitative study. JMIR Hum Factors.

[47] Ali M, Ali S, Abbas Q, Abbas Z, Lee SW (2025). Artificial intelligence for mental health: a narrative review of applications, challenges, and future directions in digital health. Digit Health.

[48] Anisha SA, Sen A, Bain C (2024). Evaluating the potential and pitfalls of AI-powered conversational agents as humanlike virtual health carers in the remote management of noncommunicable diseases: scoping review. J Med Internet Res.

[49] Laymouna M, Ma Y, Lessard D, Schuster T, Engler K, Lebouché B (2024). Roles, users, benefits, and limitations of chatbots in health care: rapid review. J Med Internet Res.

[50] Huang Y, Loux T, Huang X, Feng X (2023). The relationship between chronic diseases and mental health: a cross-sectional study. Ment Health Prev.

[51] He Y, Yang L, Qian C (2023). Conversational agent interventions for mental health problems: systematic review and meta-analysis of randomized controlled trials. J Med Internet Res.

[52] Schachner T, Keller R, V Wangenheim F (2020). Artificial intelligence-based conversational agents for chronic conditions: systematic literature review. J Med Internet Res.

[53] Hepburn J, Williams L, McCann L (2025). Barriers to and facilitators of digital health technology adoption among older adults with chronic diseases: updated systematic review. JMIR Aging.

[54] Brady J, McCloud RF, Higgins E (2025). The evolution of barriers and facilitators to using a COPD app among older adults: results from a pilot study. Front Digit Health.

[55] Yang Q, Cheung K, Zhang Y, Zhang Y, Qin J, Xie YJ (2025). Conversational agents in physical and psychological symptom management: a systematic review of randomized controlled trials. Int J Nurs Stud.

[56] Cheng W (2024). Conversational AGents: Building Trust, Enhancing User Experience, and Fostering Emotional Connections.

[57] Cheng X, Zhang X, Cohen J, Mou J (2022). Human vs. AI: understanding the impact of anthropomorphism on consumer response to chatbots from the perspective of trust and relationship norms. Inf Process Manag.

[58] Konya-Baumbach E, Biller M, von Janda S (2023). Someone out there? a study on the social presence of anthropomorphized chatbots. Comput Human Behav.

[59] Wah JNK (2025). Revolutionizing e-health: the transformative role of AI-powered hybrid chatbots in healthcare solutions. Front Public Health.

[60] Palmer CE, Marshall E, Millgate E (2025). Combining artificial intelligence and human support in mental health: digital intervention with comparable effectiveness to human-delivered care. J Med Internet Res.

[61] ter Stal S, Tabak M, op den Akker H, Beinema T, Hermens H (2020). Who do you prefer? the effect of age, gender and role on users’ first impressions of embodied conversational agents in eHealth. Int J Hum Comput Interact.

[62] Vongas JG, Al Hajj R (2015). The evolution of empathy and women’s precarious leadership appointments. Front Psychol.

[63] Nigro M, Behring GE, Aliverti A (2025). Accuracy, comprehensiveness and understandability of AI-generated answers to questions from people with COPD: the AIR-COPD Study. Respir Res.

[64] Girault A, Le A, Gonsard A (2025). ChatGPT and other large language models for parents’ questions about childhood asthma: a comparative study. Eur Respir J.

[65] Aranburu-Imatz A, López-Carrasco J de la C, Moreno-Luque A (2022). Nurse-led interventions in chronic obstructive pulmonary disease patients: a systematic review and meta-analysis. Int J Environ Res Public Health.

[66] MacNeill AL, MacNeill L, Luke A, Doucet S (2024). Health professionals’ views on the use of conversational agents for health care: qualitative descriptive study. J Med Internet Res.

[67] Lal M, Neduncheliyan S, Khurana M, Thakur A, Kantha P, Shieh CS, Shukla RK (2025). Machine Learning Algorithms.

[68] Faizan M, Han C, Lee SW (2025). Policy-driven digital health interventions for health promotion and disease prevention: a systematic review of clinical and environmental outcomes. Health Care (Don Mills).

[69] Rony MKK, Numan SM, Akter K (2024). Nurses’ perspectives on privacy and ethical concerns regarding artificial intelligence adoption in healthcare. Heliyon.

[70] Leiserowitz G, Mansfield J, MacDonald S, Jost M (2025). Patient attitudes toward ambient voice technology: preimplementation patient survey in an academic medical center. JMIR Med Inform.

[71] Asiksoy G (2025). Nurses’ assessment of artificial ıntelligence chatbots for health literacy education. J Educ Health Promot.

[72] Williamson PR, Altman DG, Blazeby JM, Clarke M, Gargon E (2011). The COMET (Core Outcome Measures in Effectiveness Trials) Initiative. Trials.

