# Supplementary material for: Conversational Agents for Asthma and Chronic Obstructive Pulmonary Disease Management: Scoping Review
Source: J Med Internet Res. 2026 Sep 29;28:e98338. doi: 10.2196/98338 (PMC13622067; doi:10.2196/98338)
Supplement: Supplementary file 1 [file jmir-v28-e98338-s001.docx]

**Supplementary File 1. Types of Conversational Agent (CA) Technology**

| **Types of Interface** | **Description (What they are and what they do)** |
| --- | --- |
| AI-based | AI-based conversational agents are a type of Large Language Model (LLM), that uses artificial intelligence, specifically Natural Language Processing (NLP) and machine learning to interpret user inputs. It can handle complex queries and are adaptable, by learning from interactions (Bilan, 2024).  Example: ChatGPT |
| Rule-based/Menu-based | Rule-based conversational agents work according to a pre-defined set of rules. It is a non-Natural Language Processing (non-NLP) model, where interactions are typically button-based, with multiple choice options. Unlike AI models, rule-based chatbots are not flexible in understanding free-text inputs but they are easy to implement (Bilan, 2024).  Example: Ask Jamie |
| Voice-based Conversational Agents (VCA) | Voice-based conversational agent uses voice commands and speech recognition to interact with users. The integration of NLP allows it to understand user commands and respond accordingly (Bilan, 2024).  Example: Apple Siri, Google Assistant, Microsoft Cortana |
| Embodied Conversational Agents (ECA) | Embodied Conversational Agents are virtual characters that imitate human behaviours and interacts with user using verbal and non-verbal cues. Due to this, they can be highly engaging and immersive for users (Provoost et al., 2017).  Example: Virtual avatar or assistant |
| Digital Twin (DT) | A digital twin is a digitised model or character that replicate human behaviour or characteristics using sensors or other sources. It can work as a conversational agent by mirroring the specific traits and characteristics of users. Hence, DTs offer personalised and customised interactions for users (Attaran & Celik, 2023) |
