# Supplementary material for: Conversational Agents for Asthma and Chronic Obstructive Pulmonary Disease Management: Scoping Review
Source: J Med Internet Res. 2026 Sep 29;28:e98338. doi: 10.2196/98338 (PMC13622067; doi:10.2196/98338)
Supplement: Supplementary file 2 [file jmir-v28-e98338-s002.docx]

. **Full Search Strategy**

| **Database: PubMed** | | |
| --- | --- | --- |
| Search Number | Search Strategy | Hits |
| #1 | “asthma”[MeSH Terms] | 146,575 |
| #2 | “asthma” [Title/Abstract] OR “respiratory sensitivity” [Title/Abstract] OR “bronchial disease*” [Title/Abstract] | 180,288 |
| #3 | #1 OR #2 | 207,651 |
| #4 | "Pulmonary Disease, Chronic Obstructive"[MeSH Terms] | 70,359 |
| #5 | “chronic obstructive pulmonary disease” [Title/Abstract] OR “copd” [Title/Abstract] OR “chronic obstructive airway disease” [Title/Abstract] OR “chronic airway obstruction” [Title/Abstract] OR “obstructive lung disease” [Title/Abstract] | 90,286 |
| #6 | #4 OR #5 | 108,473 |
| #7 | #3 OR #6 | 301,928 |
| #8 | "Artificial intelligence"[MeSH Terms] | 208,061 |
| #9 | "Artificial intelligence"[Title/Abstract] OR "natural language processing"[Title/Abstract] OR "natural language understanding"[Title/Abstract] OR "natural language generation"[Title/Abstract] OR "large language model"[Title/Abstract] OR "generative AI"[Title/Abstract] OR "chatbot"[Title/Abstract] OR "conversational agent"[Title/Abstract] OR "conversational AI"[Title/Abstract] OR "conversational artificial intelligence"[Title/Abstract] OR "conversational user interface"[Title/Abstract] OR "embodied conversational agent"[Title/Abstract] OR "conversational system"[Title/Abstract] OR "context-aware"[Title/Abstract] OR "human-computer interaction"[Title/Abstract] OR "dialog* system*"[Title/Abstract] OR "dialog* management"[Title/Abstract] OR "assist* technology"[Title/Abstract] OR "relational agent"[Title/Abstract] OR "digital agent*"[Title/Abstract] OR "digital assistant*"[Title/Abstract] OR "virtual assistant"[Title/Abstract] OR "virtual avatar"[Title/Abstract] OR "voice assistant"[Title/Abstract] OR "chatgpt"[Title/Abstract] OR "chatterbot"[Title/Abstract] | 77,746 |
| #10 | #8 OR #9 | 250,008 |
| #11 | #7 AND #10 | 1,109 |
| #12 | #11 AND (y_10[Filter]) | 916 |

| **Database: Embase** | | |
| --- | --- | --- |
| Search Number | Search Strategy | Hits |
| #1 | 'asthma'/exp OR 'chronic obstructive lung disease'/exp | 488,718 |
| #2 | "asthma*ti,ab OR 'respiratory sensitivity' ti,ab OR 'bronchial disease**.ti,ab OR 'chronic obstructive pulmonary disease': ti,ab OR 'copd':ti,ab OR 'chronic obstructive airway disease: ti,ab OR 'chronic airway obstruction':ti,ab OR 'obstructive lung disease: ti,ab | 412,336 |
| #3 | #1 OR #2 | 548,257 |
| #4 | 'chatbot'/exp OR 'artificial intelligence'/exp | 115,444 |
| #5 | 'artificial intelligence':ti,ab OR 'natural language processing':ti,ab OR 'natural language understanding':ti,ab OR 'natural language generation':ti,ab OR 'large language model':ti,ab OR 'generative ai':ti, ab OR 'chatbot':ti,ab OR 'conversational agent':ti,ab OR 'conversational ai':ti, ab OR 'conversational artificial intelligence': ti,ab OR 'conversational user interface': tiab OR 'conversational ontology': tiab OR 'embodied conversational agent':ti ab OR 'conversational system':ti ab OR 'context-aware':ti,ab OR 'human-computer interaction':ti ab OR 'dialog* system**:ti,ab OR 'dialog* management" ti,ab OR 'assist* technology' ti,ab OR 'relational agent':ti,ab OR 'digital agent*. ti,ab OR 'digital assistant**.ti,ab OR 'virtual assistant" ti,ab OR 'virtual avatar':ti,ab OR 'voice assistant':ti,ab OR 'chatgpt':ti,ab OR 'chatterbot':ti,ab | 77,717 |
| #6 | #4 OR #5 | 139,708 |
| #7 | #3 AND #6 | 1,046 |
| #8 | #7 AND [2014-2024]/py | 962 |

| **Database: CENTRAL (Cochrane Central Register of Controlled Trials)** | | |
| --- | --- | --- |
| Search Number | Search Strategy | Hits |
| #1 | MeSH descriptor: [Asthma] explode all trees | 15089 |
| #2 | MeSH descriptor: [Pulmonary Disease, Chronic Obstructive] explode all trees | 8331 |
| #3 | "asthma" OR "respiratory sensitivity" OR (bronchial NEXT disease*) OR "Chronic obstructive pulmonary disease" OR "copd" OR "chronic obstructive airway disease" OR "chronic airway obstruction" OR "obstructive lung disease" | 61679 |
| #4 | #1 OR #2 OR #3 | 62375 |
| #5 | MeSH descriptor: [Artificial Intelligence] explode all trees | 3279 |
| #6 | "Artificial intelligence" OR "natural language processing" OR "natural language understanding" OR "natural language generation" OR "large language model" OR "generative Al" OR "chatbot" OR "conversational agent" OR "conversational Al" OR "conversational artificial intelligence" OR "conversational user interface" OR  "conversational ontology" OR "embodied conversational agent" OR "conversational system" OR "context aware" OR "human computer interaction" OR (dialog* NEXT system*) OR (dialog* NEXT management) OR (assist* NEXT technology) OR "relational agent" OR "digital agent" OR "digital assistant" OR "virtual assistant" OR  "virtual avatar" OR "voice assistant" OR "chatapt" OR "chatterbot" | 4024 |
| #7 | #5 OR #6 | 6466 |
|  | #4 AND #7 with Cochrane Library publication date from Jan 2014 to Dec 2024 | 62 |

| **Database: Web Of Science** | | |
| --- | --- | --- |
| Search Number | Search Strategy | Hits |
| #1 | TS=("asthma" OR "respiratory sensitivity" OR "bronchial disease*") | 241,017 |
| #2 | TS=("Chronic obstructive pulmonary disease" OR "copd" OR "chronic obstructive airway disease" OR "chronic airway obstruction" OR "obstructive lung disease") | 117,509 |
| #3 | #1 OR #2 | 338,874 |
| #4 | TS=(“Artificial intelligence” OR “natural language processing” OR “natural language understanding” OR “natural language generation” OR “large language model” OR “generative AI” OR “chatbot” OR “conversational agent” OR “conversational AI” OR “conversational artificial intelligence” OR “conversational user interface” OR “conversational ontology” OR "embodied conversational agent" OR "conversational system" OR “context-aware” OR “human-computer interaction” OR "dialog* system*" OR “dialog* management” OR "assist* technology" OR "relational agent" OR "digital agent*" OR "digital assistant*" OR "virtual assistant" OR “virtual avatar” OR "voice assistant" OR "chatgpt" OR “chatterbot”) | 299,241 |
| #5 | #3 AND #4 | 559 |
| #6 | #3 AND #4 and 2014 or 2015 or 2016 or 2017 or 2018 or 2019 or 2020 or 2021 or 2022 or 2023 or 2024 (Publication Years) | 515 |

| **Database: CINAHL** | | |
| --- | --- | --- |
| Search Number | Search Strategy | Hits |
| S1 | (MH asthma) OR (MH Pulmonary Disease, Chronic Obstructive)  Expanders - Apply equivalent subjects  Search modes - Find all my search terms | 58,153 |
| S2 | TI ( “asthma” OR “respiratory sensitivity” OR “bronchial disease*” OR “Chronic obstructive pulmonary disease” OR “copd” OR “chronic obstructive airway disease” OR “chronic airway obstruction” OR “obstructive lung disease” ) OR AB ( “asthma” OR “respiratory sensitivity” OR “bronchial disease*” OR “Chronic obstructive pulmonary disease” OR “copd” OR “chronic obstructive airway disease” OR “chronic airway obstruction” OR “obstructive lung disease” )  Expanders - Apply equivalent subjects  Search modes - Find all my search terms | 66,308 |
| S3 | S1 OR S2  Expanders - Apply equivalent subjects  Search modes - Find all my search terms | 79,254 |
| S4 | (MH artificial intelligence)  Expanders - Apply equivalent subjects  Search modes - Find all my search terms | 13,939 |
| S5 | TI ( “Artificial intelligence” OR “natural language processing” OR “natural language understanding” OR “natural language generation” OR “large language model” OR “generative AI” OR “chatbot” OR “conversational agent” OR “conversational AI” OR “conversational artificial intelligence” OR “conversational user interface” OR “conversational ontology” OR "embodied conversational agent" OR "conversational system" OR “context-aware” OR “human-computer interaction” OR "dialog* system*" OR “dialog* management” OR "assist* technology" OR "relational agent" OR "digital agent*" OR "digital assistant*" OR "virtual assistant" OR “virtual avatar” OR "voice assistant" OR "chatgpt" OR “chatterbot” ) OR AB ( “Artificial intelligence” OR “natural language processing” OR “natural language understanding” OR “natural language generation” OR “large language model” OR “generative AI” OR “chatbot” OR “conversational agent” OR “conversational AI” OR “conversational artificial intelligence” OR “conversational user interface” OR “conversational ontology” OR "embodied conversational agent" OR "conversational system" OR “context-aware” OR “human-computer interaction” OR "dialog* system*" OR “dialog* management” OR "assist* technology" OR "relational agent" OR "digital agent*" OR "digital assistant*" OR "virtual assistant" OR “virtual avatar” OR "voice assistant" OR "chatgpt" OR “chatterbot” )  Expanders - Apply equivalent subjects  Search modes - Find all my search terms | 18,402 |
| S6 | S4 OR S5  Expanders - Apply equivalent subjects  Search modes - Find all my search terms | 25,578 |
| S7 | S3 AND S6  Expanders - Apply equivalent subjects  Search modes - Find all my search terms | 94 |
| S8 | S7  Limiters - Publication Date: 20140101- 20241231  Expanders - Apply equivalent subjects Search modes - Find all my search terms | 74 |

| **Database: IEEE Xplore** | | |
| --- | --- | --- |
| Search Number | Search Strategy | Hits |
| #1 | ("All Metadata"."asthma" OR "AlI Metadata" "respiratory sensitivity" OR "All Metadata":"bronchial disease*") OR ("All Metadata":"chronic obstructive pulmonary disease" OR "All Metadata". "copd" OR "All Metadata":"chronic obstructive airway disease" OR "All Metadata" "chronic airway obstruction" OR "AIl Metadata":"obstructive lung disease") | 1843 |
| #2 | ("All Metadata" "artificial intelligence" OR "AII Metadata" "natural language processing" OR "All Metadata" "natural language understanding" OR "All Metadata" :"natural language generation" OR "All  Metadata" :"large language model" OR "All Metadata" :"generative Al" OR "All Metadata":"chatbot" OR "All Metadata" "conversational agent" OR "All Metadata":"conversational Al" OR "AlI Metadata" :"conversational artificial intelligence" OR "All Metadata" "conversational user interface" OR "All Metadata": "conversational ontology" OR "All Metadata" "embodied conversational agent" OR "All Metadata": "conversational system" OR "All Metadata" ."context-aware" OR "All Metadata" "human-computer interaction" OR "All Metadata" ."dialog* system*" OR "AlI Metadata" :"dialog* management" OR "AlI Metadata" "assist* technology" OR "All Metadata" "relational agent" OR "AlI Metadata":"digital agent*" OR "AlI Metadata" "digital assistant*" OR "All Metadata" "virtual assistant*" OR "All Metadata" :"virtual avatar*" OR "All Metadata" :"voice assistant' OR "All Metadata" "chatgpt" OR "All Metadata" "chatterbot") | 301,742 |
| #3 | #1 AND #2  You Refined By: Year: 2014-2024 | 853 |

| **Database: Scopus** | | |
| --- | --- | --- |
| Search Number | Search Strategy | Hits |
| #1 | TITLE-ABS-KEY ("asthma" OR "respiratory sensitivity" OR "bronchial disease*") | 319,668 |
| #2 | TITLE-ABS-KEY ("chronic obstructive pulmonary disease" OR "copd" OR "chronic obstructive airway disease" OR "chronic airway obstruction" OR "obstructive lung disease") | 173,559 |
| #3 | #1 OR #2 | 457,543 |
| #4 | TITLE-ABS-KEY ("artificial intelligence" OR "natural language processing" OR "natural language understanding" OR "natural language generation" OR "large language model" OR "generative a¡" OR "chatbot" OR "conversational agent" OR "conversational ai" OR "conversational artificial intelligence" OR "conversational user interface" OR "conversational ontology" OR "embodied conversational agent" OR "conversational system" OR  "context-aware" OR "human-computer interaction" OR "dialog* system*" OR "dialog* management" OR "assist* technology" OR "relational agent" OR "digital agent*" OR "digital assistant*" OR "virtual assistant" OR "virtual avatar" OR "voice assistant" OR "chatgpt" OR "chatterbot") | 904,797 |
| #5 | #3 AND #4 | 1,415 |
| #6 | #5 AND PUBYEAR > 2013 AND PUBYEAR < 2025 | 1,240 |

| **Database: PROQUEST THESIS AND DISSERTATIONS** | | |
| --- | --- | --- |
| Search Number | Search Strategy | Hits |
| S1 | noft("asthma" OR "respiratory sensitivity" OR "bronchial disease*") | 8,948 |
| S2 | noft("chronic obstructive pulmonary disease" OR "copd" OR "chronic obstructive airway disease" OR "chronic airway obstruction" OR "obstructive lung disease") | 4,395 |
| S3 | S1 OR S2 | 12,444 |
| S4 | noft(“Artificial intelligence” OR “natural language processing” OR “natural language understanding” OR “natural language generation” OR “large language model” OR “chatbot” OR “conversational agent” OR “conversational user interface” OR “conversational ontology” OR "embodied conversational agent" OR "conversational system" OR "dialog* system*" OR “dialog* management” OR "assistance technology" OR "relational agent" OR "digital agent*" OR "digital assistant*" OR "virtual assistant" OR "voice assistant" OR "chatgpt" OR "chatterbot") | 64,646 |
| S5 | S3 AND S4 | 105 |
| S6 | S5 AND yr(2014-2024) | 93 |

| **Clinical Registry: WHO ICTRP** | | |
| --- | --- | --- |
| Search Number | Search Strategy | Hits |
| #1 | “asthma” OR “respiratory sensitivity” OR “bronchial disease*” OR “Chronic obstructive pulmonary disease” OR “copd” OR “chronic obstructive airway disease” OR “chronic airway obstruction” OR “obstructive lung disease” [in the Condition]  AND  “Artificial intelligence” OR “natural language processing” OR “natural language understanding” OR “natural language generation” OR “large language model” OR “generative AI” OR “chatbot” OR “conversational agent” OR “conversational AI” OR “conversational artificial intelligence” OR “conversational user interface” OR “conversational ontology” OR "embodied conversational agent" OR "conversational system" OR “context-aware” OR “human-computer interaction” OR "dialog* system*" OR “dialog* management” OR "assist* technology" OR "relational agent" OR "digital agent*" OR "digital assistant*" OR "virtual assistant" OR “virtual avatar” OR "voice assistant" OR "chatgpt" OR “chatterbot” [in the Intervention]  Recruitment status is: ALL  Date of registration is between: 01/01/2014 and 31/12/2024 | 3 |

| **Clinical Registry: CLINICALTRIALS.GOV** | | |
| --- | --- | --- |
| No. | Search Terms | Hits |
| #1 | “asthma*” OR “respiratory sensitivity” OR “bronchial disease*” OR “Chronic obstructive pulmonary disease” OR “copd” OR “chronic obstructive airway disease” OR “chronic airway obstruction” OR “obstructive lung disease” AND  “Artificial intelligence” OR “natural language processing” OR “natural language understanding” OR “natural language generation” OR “large language model” OR “generative AI” OR “chatbot” OR “conversational agent” OR “conversational AI” OR “conversational artificial intelligence” OR “conversational user interface” OR “conversational ontology” OR "embodied conversational agent" OR "conversational system" OR “context-aware” OR “human-computer interaction” OR "dialog* system*" OR “dialog* management” OR "assist* technology" OR "relational agent" OR "digital agent*" OR "digital assistant*" OR "virtual assistant" OR “virtual avatar” OR "voice assistant" OR "chatgpt" OR “chatterbot”  AND  Study start from 01/01/2014 to 12/31/2024 | 39 |
