# Supplementary material for: Conversational Agents for Asthma and Chronic Obstructive Pulmonary Disease Management: Scoping Review
Source: J Med Internet Res. 2026 Sep 29;28:e98338. doi: 10.2196/98338 (PMC13622067; doi:10.2196/98338)
Supplement: Supplementary file 3 [file jmir-v28-e98338-s003.docx]

| **Author/Year** | **Roles** | | | **Direct patient outcomes** | | **Indirect patient outcomes** | |
| --- | --- | --- | --- | --- | --- | --- | --- |
|  | **Disseminating information and patient education** | **Collect user data and provide disease support** | **Provide emotional and psychosocial support** | **Clinical outcomes** | **Behavioural changes** | **User experience** | **User preference** |
| Alabdulmohsen et al., 2024 | Reliable as source of information  Answers evaluated by 5 board-certified internal medicine physicians for reliability and acceptability  93.33% responses had good reliability  78.3% acceptable to be the only answers for an asthmatic patient  2/30 questions had inconsistent answers | N/A | N/A | N/A | N/A | Readability was difficult | N/A |
| Cleres et al., 2021 | N/A | useful to give daily assessment on COPD control study | N/A | N/A | N/A | Perceived ease of use, perceived enjoyment, intention to use, perceived usefulness: scored between “agree” to “strongly agree”  Interacting verbally increase compliance as it is more engaging (n = 1)  Lena took too long to interpret question, and speech and intonation of Lena was a disadvantage, making dialogue sluggish sometimes (n = 2) | Relative advantage of using Lena than filling in questionnaire on smartphone: scored between “neutral” and “agree”  Will not fit in luggage while traveling (n = 1)  System needs to be plugged in, will not work in power outage (n = 1)  Preference for dialect (n = 2) |
| Cook et al., 2024 | asthma health literacy education via conversations about disease, proper medication use, potential triggers and management strategies | - assessment of asthma risk within next 3 months via a novel risk model  - assess asthma control over time via the Asthma Control Questionnaire  - Generate report of interaction for discussion with healthcare professionals | - personalised encouragement to access basic healthcare services customised to their levels of risk and control | After 28-day interaction  - 8.38% better on Asthma Control Questionnaire compared to Day 0 and was clinically significant | - 45.59% agreed to wear a scarf during cold weather  - 30.77% to replace carpet with hard flooring  - 33.33% to keep pets out of bedroom  - 66.67% to check pollen forecast | Helpful (80% agreed), private and secure (87%), trustworthy (80%), enjoyable (68%) and bug free (84%)  In the third iteration of the CA, only 27% (14/52) said they  would recommend Brisa to a friend with asthma, showing a  decline in user satisfaction since Wave 2 (despite having provided improvements based on feedback in Wave 2) | Demand for greater conversational scope and fluency  - “the conversation was two-way” (49% agreed)  - “the conversation felt personalised” (46% agreed)  - “Brisa provided enough information” (48% agreed) |
| Easton et al. 2019 | N/A | Useful for those who do not want to burden partners (n = 1)  Positive about using Ava to support them during an acute COPD exacerbation | Provide motivational support and particularly useful for those living alone (n = 5) | N/A | N/A | System usability score (median = 73.75)  50% strongly agreed that they would use the system frequently, 50% unsure  88% strongly agreed that system was easy to use  All but 1 strongly agreed that they would feel confident using the system  Mixed findings on if they needed a technical support person, and whether they needed to learn a lot of use the system  The group thought it would be particularly useful for those living alone or those uncomfortable with burdening others | Ava should be available on different electronic devices (n = 9)  Be able to use text and speech to communicate (n = 3)  Internet-of-things applications (n = 3)  Encourage user to call emergency services when appropriate |
| Gonsard et al., 2023 | N/A | Children’s perspectives  - Remote asthma management (47%)  - Assistance with daily asthma management (27%)  - Management in real time (26%)  - Better performance than physicians (9%), lower performance than physicians (18%)  - Decreased dependence on physician (8%)  - Risks related to personal data needed to run system (11%) | N/A | N/A | N/A | Children’s perspectives  - 14% didn’t find it beneficial  - No idea of any disadvantage (n = 43%), no concerns (n = 55%)  - Technical issues related to embedded AI (n = 53%)  - Loss of humanity (24%)  - Risks related to connected objects required (physical harm and fragility) (5%)  - Negative impact on development of autonomy | Seven percent never chose the system, even when its hypothetical ability to control asthma was 30% better than that of the physician alone.  If the system was similarly effective as the physician, 56% of participants would use it, and 93% if it was 30% more effective.  Even when the system was considered 10% less effective than the physician, 23% of children preferred this new type of care. These results were not different between children (8–12 years old) and adolescents (13–17 years old) (p = 0.6).  31% of parents would never choose the system. 43% were willing if it was as effective as the physician, and up to 69% if the DTS was 30% better than the physician’s usual management.  Children were more prone to choose the system-based management if they had high computer skills and less prone if they valued the physician–patient relationship  In terms of medical decisions about themselves, most of the children (19%) would still prefer that medical decisions about them are only based on their doctor. |
| Gross et al., 2021 | N/A | N/A | N/A | N/A | Those who interacted with paternalistic CA more willing to change their behaviour compared to a deliberative CA | Older patients reported better relationship quality with CA than younger ones, irrespective of CA type  Those with more severe COPD reported better relationship than those with less severe COPD, irrespective of CA type  Those with higher disease literacy of COPD had better working alliance irrespective of assigned CA type | Severe COPD patients preferred paternalistic conversational style  Younger patients and those with less recent diagnosis preferred the deliberative interaction style  The higher the GOLD classification and those with fewer years of experience of COPD, the more likely they intend to continue with the CA  Older patients more likely to continue when working with a paternalistic CA, while younger preferred a deliberative CA |
| Høj et al., 2024 | ChatGPT had high accuracy: 81% scored 4/5 or higher | N/A | N/A | N/A | N/A | Readability was difficult | N/A |
| Kadariya et al., 2019 | N/A | N/A | N/A | N/A | N/A | Overall technology acceptance value >8 on 11-point Likert scale  Mean system usability score: 83.13 for clinicians, 82.81 for researchers | N/A |
| Kohlbrenner et al., 2024 | Living with COPD educational program delivered virtually via the CA, addressing comprehensive topics tailored for patients with COPD, ranging from coping during activities of daily living to travel advice  Interactions were self-paced with predefined answer options | ✓ | N/A | - Health impairment in COPD (St. George Respiratory Questionnaire): improved in both groups without statistical or clinically significant between-group differences  - Symptom burden (COPD Assessment Test) decreased in both groups without statistical but clinically significant between-group differences.  - Functional exercise capacity (6-min walk test and 1-min sit-to-stand test): improved for both groups but no statistical significance between-group difference  - Lung function (spirometry) remained stable for both groups | - Physical activity (daily step count): increased only in intervention group without statistically significant but clinically relevant between-group differences | Most were not bothered by the daily sensor interactions. Sensors that required minimal or no interaction (cough reading and physical activity monitor) scored best | N/A |
| Kowatsch et al., 2021 | Quizzes and educational video clips | ✓ | Supports family members | Significant increase in asthma knowledge | Healthcare professionals identified 2/42 inhalation video clips submitted to have serious mistakes and subsequently the corrected video clips submitted had no severe inhalation mistakes. | - Liked educational content of intervention and text-based features the most  - 49.5% of young patients did install the app and started to interact with the MAX ca. | - Patients and family members felt there was too much predefined text  - Lack of access to smartphones, especially young patients was a limiting factor to further increase reach of intervention  - Healthcare professionals preferred to also include younger and older patients and to integrate further interaction between healthcare professionals and patients |
| Quinde et al., 2020 | N/A | Triggers assistance, control test management, communication, personalised monitoring, medication reminder assessed as very useful | N/A | N/A | N/A | Virtual assistant was rated between useful and very useful and the least important feature  Virtual assistant feature is helpful for those familiar with it and those who want it, but only one uses them regularly.  Virtual assistant does not recognise their accent | Ask the Asthma Control Questionnaire through the virtual assistant in order to have a better-quality assessment, (n = 1)  Define a phrase that the virtual assistant can recognise as emergency (n = 2)  Use it to record a  diary of the triggers and symptoms they experience (n = 1)  What the participants would prefer to use in order to keep track of their asthma health status.  - virtual assistant only (n = 1)  - Use the Asthma Control Assessment Component only (n = 2)  - use both options depending on the situation (n = 7) |
| Rhee et al., 2014 | N/A | improved awareness of symptoms and triggers | - promoted treatment adherence and sense of control  - facilitated adolescent–parent partnerships. | N/A | Improved asthma self-management and medication adherence | Could contribute to effective asthma self-management  Technical challenges especially in beginning of trial e.g. establishing intial connection with system, often when participants used prepaid phone services  Challenges associated with system’s limited lexicon dictionary that often triggered the system’s automatic response of “I don’t understand” | Expand system’s language-recognition capacity and maximizing flexibility by allowing users control timing of interactions with the system  Parents: Features to facilitate information sharing with healthcare providers could further strengthen its clinical usefulness  System could be more beneficial and helpful for those whose asthma is not controlled. |
| Rodrigues et al., 2022 | N/A | N/A | N/A | N/A | N/A | Easy to use the voice to  complete the tasks for usability testing and system could handle their accent well. However, some users also reported that the chatbot didn’t understand some words user said.  Mainly the younger users reported that they would like to perform actions in one simple phrase, like ‘Change my name to Richard’, and that the chatbot could talk in a more casual way, not formally.  Good chatbot to interact with, it can help in multiple ways, and that it is fun to talk with.  Chatbot takes too much time to answer | Preferred that chatbot speak in Portuguese instead of Brazilian Portuguese |
| Sloots et al., 2021 | CA provided recommendations to promote healthy behaviour | CA gave reminders on completing to-do list, feedback on inhaled medication adherence and technique | N/A | (no statistical testing reported)  - COPD Self-efficacy Score decreased (mean, SD): 75.2 (26.6) to 73.9 (22.8)  - COPD self-management behaviour and knowledge (Partners in Health question) (mean, SD): 81.7 (8.1) to 86.4 (4.0)  - Health-related quality of life (St. George Respiratory Questionnaire) decreased (median, IQR): 45.0 (9.2-68.2) to 43.1 (6.9-64.0)  - Hospital Anxiety Depression score (anxiety) no change (median, IQR): 5 (2-10) to 5 (0-12)  - Hospital Anxiety Depression score (depression) decreased (median, IQR): 5 (1-10) to 4 (1-9) | Adherence to completing daily diaries was high  Advised actions were inadequately followed up, especially “call the case manager”  Inhaled medication adherence was high but inhalations were poorly performed | Use of home monitoring devices led to connection and low-battery problems. Due to system failures, the application could not be used for 2 days. With other technical issues, it led to some frustrations and distrust amongst patients regarding eHealth self-management intervention | N/A |
| ter Stal et al., 2021 | Same as Sloots et al., 2021 | Same as Sloots et al., 2021 | N/A | N/A | Less likely to follow the agent’s advice (p=0.01) | Not seen as reliable: gave tips that did not fit their individual situation, another participant called the agent “a stupid woman”  Mixed opinions on agent’s small talk, with some not being interested in it (n = 5)  Mixed opinions on cartoon appearance of the agent with some preferring it to look more like a healthcare professional  Found it annoying as it provided too many messages or that they were sent at inappropriate times and that the agent provided unwanted and unsolicited information  Agent did not respond to user input, so did not find advice useful, another will visit the doctor to verify first. One said that they will not listen to a cartoon figure.  Did not think that it had any value (n = 7): statements were too general and simplistic, and clear that they were not personalised | Preferred to be able to text a question and receive a response 24/7 (n = 2) |
| Wegener et al., 2024 | Knowledge or management of lung capacity to learn about what they can do depending on their condition  Ideas and help to fall asleep for those who need it | Option to share data with healthcare professionals and informal caregivers, but some valued privacy more | N/A | N/A | N/A | N/A | Not interested in a technology to replace contact with healthcare professionals.  Open to reading health-related information from trusted sources  Mixed findings on extent and frequency of measurements to monitor their condition  Daily activity function to suggest social activities in local area and monitor physical activity through step count for example |

CA (conversational agent), COPD (chronic obstructive pulmonary disease), N/A (not applicable)

## REFERENCES

1. Alabdulmohsen DM, Almahmudi MA, Alhashim JN, et al. Is ChatGPT a reliable source of patient information on asthma? Cureus. Jul 2024;16(7):e64114.
2. Cleres DM, Rassouli F, Brutsche M, Kowatsch T, Barata F. Lena: a voice-based conversational agent for remote patient monitoring in chronic obstructive pulmonary disease. ETH Zurich; Apr 13, 2021.
3. Cook D, Peters D, Moradbakhti L, et al. A text-based conversational agent for asthma support: mixed-methods feasibility study. Digit Health. 2024;10:20552076241258276.
4. Easton K, Potter S, Bec R, et al. A virtual agent to support individuals living with physical and mental comorbidities: co-design and acceptability testing. J Med Internet Res. May 30, 2019;21(5):e12996.
5. Gonsard A, AbouTaam R, Prévost B, et al. Children's views on artificial intelligence and digital twins for the daily management of their asthma: a mixed-method study. Eur J Pediatr. Feb 2023;182(2):877-888.
6. Gross C, Schachner T, Hasl A, et al. Personalization of conversational agent-patient interaction styles for chronic disease management: two consecutive cross-sectional questionnaire studies. J Med Internet Res. May 26, 2021;23(5):e26643.
7. Høj S, Thomsen SF, Ulrik CS, Meteran H, Sigsgaard T, Meteran H. Evaluating the scientific reliability of ChatGPT as a source of information on asthma. J Allergy Clin Immunol Glob. Nov 2024;3(4):100330.
8. Kadariya D, Venkataramanan R, Yip HY, Kalra M, Thirunarayanan K, Sheth A. KBot: knowledge-enabled personalized chatbot for asthma self-management. Presented at: Proceedings of the 2019 IEEE International Conference on Smart Computing (SMARTCOMP). Jun 12-14, 2019:IEEE. 138-143; Washington, DC, USA. 2019.
9. Kohlbrenner D, Kuhn M, Kläy A, et al. Hybrid virtual coaching and telemonitoring in COPD management: The Cair randomised controlled study. Int J Chron Obstruct Pulmon Dis. 2024;19:2739-2750.
10. Kowatsch T, Schachner T, Harperink S, et al. Conversational agents as mediating social actors in chronic disease management involving health care professionals, patients, and family members: multisite single-arm feasibility study. J Med Internet Res. Feb 17, 2021;23(2):e25060.
11. Quinde M, Augusto JC, Khan N, van Wyk A. ADAPT: approach to develop context-aware solutions for personalised asthma management. J Biomed Inform. Nov 2020;111:103586.
12. Rhee H, Allen J, Mammen J, Swift M. Mobile phone-based asthma self-management aid for adolescents (mASMAA): a feasibility study. Patient Prefer Adherence. 2014;8:63-72.
13. Rodrigues R, Caceiro R, Brites-Pereira M, et al. A conversational agent for promoting physical activity among COPD patients. In: Rocha A, Adeli H, Dzemyda G, Moreira F, editors. Information Systems and Technologies. Springer International Publishing; 2022:486-497. ISBN: 978-3-031-04825-8.
14. Sloots J, Bakker M, van der Palen J, et al. Adherence to an eHealth self-management intervention for patients with both COPD and heart failure: results of a pilot study. Int J Chron Obstruct Pulmon Dis. 2021;16:2089-2103.
15. Ter Stal S, Sloots J, Ramlal A, Op den Akker H, Lenferink A, Tabak M. An embodied conversational agent in an eHealth self-management intervention for chronic obstructive pulmonary disease and chronic heart failure: exploratory study in a real-life setting. JMIR Hum Factors. Nov 4, 2021;8(4):e24110.
16. Wegener EK, M Bergschöld J, Kramer T, Schmidt CW, Borgnakke K. Co-designing a conversational agent with older adults with chronic obstructive pulmonary disease who age in place: qualitative study. JMIR Hum Factors. Oct 8, 2024;11:e63222.
